# Oblique lateral interbody fusion (OLIF) compared with unilateral biportal endoscopic lumbar interbody fusion (ULIF) for degenerative lumbar spondylolisthesis: a 2-year follow-up study

**DOI:** 10.1186/s13018-023-04111-x

**Published:** 2023-08-24

**Authors:** Shuyan Cao, Bingjie Fan, Xin Song, Yi Wang, Wenzhe Yin

**Affiliations:** 1 Department of Orthopaedics, The Second Affiliated Hospital of Harbin Medical University, Harbin, Heilongjiang China; 2 Department of Oncology, Affiliated Hospital of Guizhou Medical University, Guiyang, Guizhou China; 3 Department of Orthopaedics, The First Affiliated Hospital of Zhengzhou University, Zhengzhou, Henan China

**Keywords:** OLIF-AF, ULIF; DLS, Clinical efficacy, Radiological results

## Abstract

**Background:**

Oblique lumbar interbody fusion (OLIF) has been proven to be an effective method of indirect decompression for the treatment of Degenerative Lumbar Spondylolisthesis (DLS). However, its superiority over Unilateral biportal endoscopic Lumbar Interbody Fusion (ULIF) has not been reported yet. The current study aimed to compare the clinical and radiological outcomes of OLIF and ULIF in patients with DLS.

**Methods:**

A total of 107 patients were included in this study, divided into two groups according to the surgical methods with 45 patients treated by OLIF combined with anterolateral single screwrod fixation, and 62 patients treated by ULIF. To compare the perioperative parameters (blood loss, operation time, and postop hospitalization) and clinical (the Visual Analog Scale (VAS) scores of the low back pain and leg pain and the Oswestry Disability Index (ODI)) and radiological (disk height (DH), lumbar lordosis (LL), segmental lordosis (SL), the cross-sectional area (CSA) of the spinal canal) results of the two surgical approaches to evaluate their efficacy.

**Results:**

Compared with the ULIF group, the blood loss and operation time in the OLIF-AF group were significantly reduced, and the Postop hospitalization was comparable. The VAS scores in both groups were significantly improved compared to preop; however, the VAS score of low back pain in the OLIF-AF group was superior to that in ULIF group throughout the follow-up period (*P* < 0.05). The improvements in DH, LL, and Segmental angle were significantly lower in the ULIF group, and the expansion rate of CSA in the OLIF-AF group was superior to that in the ULIF group, but the difference was not statistically significant. The fusion rate in OLIF-AF group was significantly higher than that in ULIF group within 6 mo postop, and there was no significant difference at the last follow-up. The incidence of complications was comparable between the two groups, and there was no statistical difference.

**Conclusions:**

Both OLIF-AF and ULIF achieved good short-term results in the treatment of DLS, and both surgical approaches are desirable. However, OLIF-AF has advantages over ULIF in terms of postoperative restoration of lumbar sagittal parameters and earlier intervertebral fusion. Long-term follow-up and larger clinical studies are needed to confirm this result.

## Introduction

Currently, with the increasing aging of the global population, the prevalence of degenerative lumbar spondylolisthesis (DLS) is approximately 5–7%, and it is also a common cause of low back pain in middle-aged and elderly people, often combined with intermittent claudication and cauda equina syndrome, which seriously affects the quality of life of patients [1]. The etiology of DLS is complex, according to previous studies, and is often associated with disk degeneration, small joint osteoarthritis, altered hormone levels, and spinal stenosis [2]. Surgical intervention is often required when satisfactory results cannot be obtained with conservative treatment [3].

Lumbar interbody fusion (LIF) has been recognized as an established treatment for intermittent claudication due to intervertebral instability [4]. Through the rapid development of surgical techniques as well as endoscopic techniques in recent decades, a variety of surgical modalities have been discovered. In ULIF, two working channels are established so that the observation channel and the operation channel are separated from each other and do not hinder each other. This not only combines the advantages of an open surgical field of vision and a large operating range but also avoids the damage of minimally invasive TLIF technology to the muscle-ligament structure due to the use of a tubular retractor and can also achieve direct decompression through unilateral discectomy, facetectomy, and bilateral laminoforaminotomy via a unilateral approach bilateral intervertebral foramen incision and intervertebral fusion under direct vision [5, 6]. To minimize the trauma associated with posterior surgery, ALIF, as well as LLIF, were introduced, but since they both have their limitations, to circumvent these problems, Silvestre et al. [7] first reported oblique lateral interbody fusion (OLIF) in 2012, the OLIF reaches the intervertebral space through the gap between the retroperitoneal fat and the psoas major muscle to perform an indirect decompression of the spinal canal to perform indirect decompression and reconstruct spinal stability [8].

Previous studies on the OLIF for DLS are scarce, especially when compared with the ULIF, so we conducted this study to compare the short-term clinical and radiological outcomes of OLIF combined with anterolateral single screwrod fixation (OLIF-AF group) and the ULIF for DLS.

## Methods

### Ethics

This retrospective study was approved by the hospital ethics committee, approval number: 2022KY0771002. All patients signed a preoperative informed consent form for surgery. The selection of OLIF-AF and ULIF was agreed upon by the spine surgeons in our department after discussion with the patients, and all procedures were performed by the same senior spine surgeon.

### Inclusion and exclusion criteria

The inclusion criteria were: (1) Recurrent lumbosacral pain with or without intermittent claudication; (2) Diagnosis of single-segment Meyerding I or II degree vertebral slippage (L2/3, L3/L4 or L4/L5) on radiological; (3) No significant improvement in symptoms after 3–6 months of regular conservative treatment with a clear diagnosis;

The exclusion criteria were: (1) history of previous lumbar spine surgery; (2) spinal infection and tumor; (3) combined lateral kyphosis deformity; (4) combined with multiple underlying diseases that cannot tolerate surgery. (5) Combined cauda equina syndrome.

### Patient data

Patients who came to our department for LIF from June 2019 to September 2021 were collected, and patients were enrolled according to the above inclusion as well as exclusion criteria, and their general data were recorded, mainly including gender, age, BMI, slippage grading, and follow-up time.

### Surgical techniques

#### Surgical techniques of OLIF-AF

**Fig. 1:**
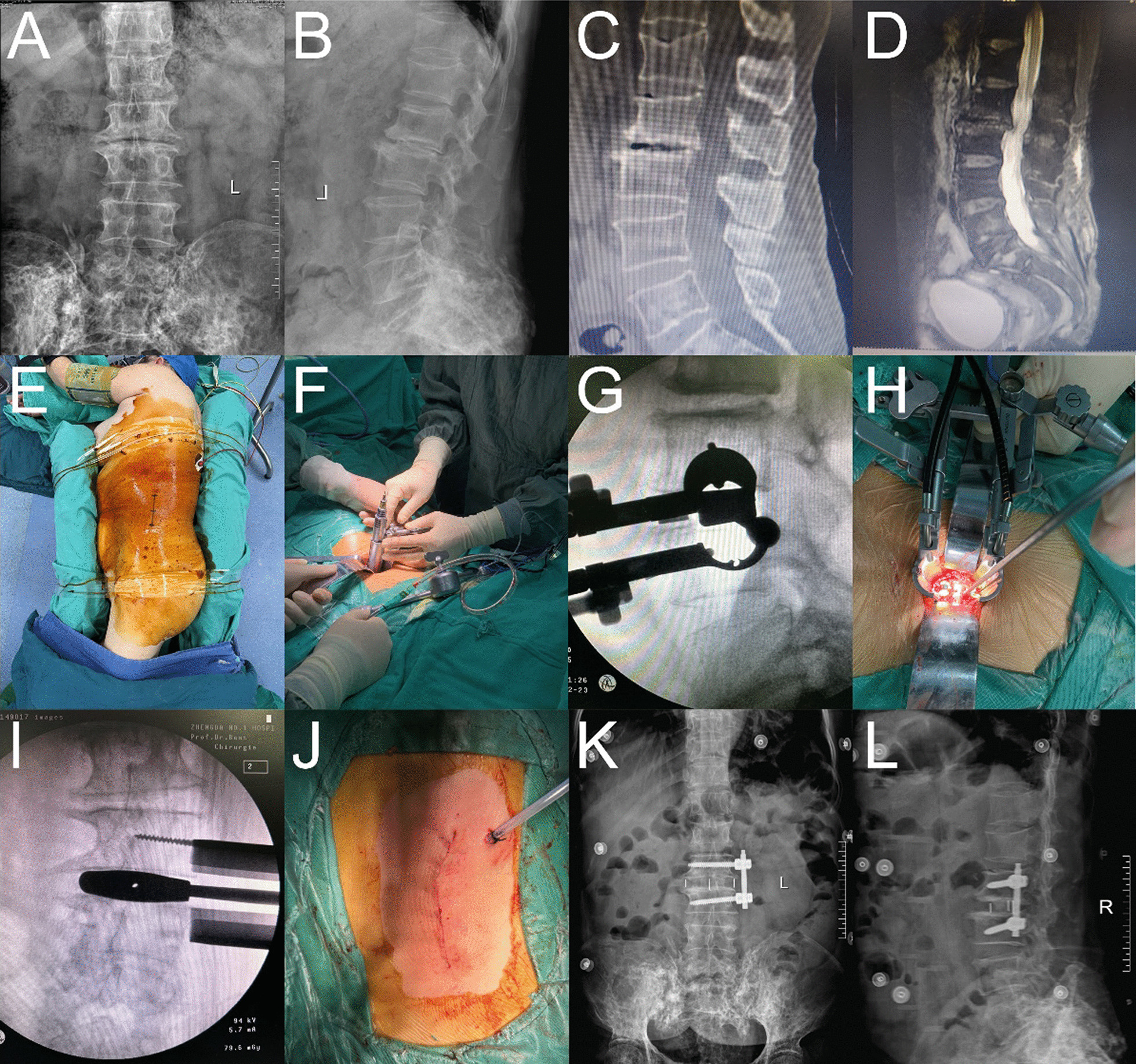
OLIF-AF surgical procedure. **A**, **B**: DR in preop; **C**: CT in preop; **D**: MRI in preop; **E**: marked the body surface; **F**: insert the sleeve; **G**: confirmed the intervertebral space; **H**: endplate preparation; **I**: implanted cages; **J**: sutured the incision; **K**, **L**: DR in postop reexamination

Taking L2/3 as an example, DR (Fig. 1A, B), CT (Fig. 1C), and MRI (Fig. 1D) in preop as follows. After successful general anesthesia, the patient was placed on the right lateral decubitus, and the body surface was marked under fluoroscopic guidance before routine disinfection (Fig. 1E). Cut the skin, the retroperitoneal space was accessed by blunt dissection after abdominal muscles separated along their fibers. The fascia of the psoas muscle was stripped away from the ventral side of the belly of psoas muscle, then insert the sleeve (Fig. 1F). After the distractor was placed, the intervertebral space (Fig. 1G) was confirmed by a C-arm fluoroscopic examination. After the light source was connected, the intervertebral disks were processed and endplate preparation (Fig. 1H) was conducted. After the model test, the appropriate cage (Fig. 1I) was implanted, the size of the cage is 45–55 mm long, 18–22 mm wide and 10–14 mm high. Meanwhile, artificial bone was filled in to fix the lateral screw of the vertebral body. After adequate hemostasis, the incision (Fig. 1J) was sutured. Postop reexamination showed that the internal fixation position of DR (Fig. 1K, L) was good, and the lumbar spondylolisthesis had been fully reduced.

#### Surgical techniques of ULIF

After successful general anesthesia, the patient was placed in the prone position. Positioning responsible segments and bilateral pedicle surface projections under C-arm fluoroscopy were marked on the skin. Routine disinfection, centered on the surgical incision, ensured the smooth flow of lavage fluid out of the surgical area. A guidewire was inserted into each pedicle, and two transverse incisions of about 1–2 cm were made at the projection of the superior and inferior pedicles on the side with severe symptoms. The observation channel was located on the left side of the operator, and the operation channel was located on the right side of the operator. The skin, subcutaneous tissue, and deep fascia were dissected in layers, and the muscle tissue on the surface of the lamina and spinous process was bluntly separated to enlarge the entry of the instrument. After connecting the endoscopic system (KARL STORZ Company, IMAGE1 S camera system), a radiofrequency tool was used for further exposure of the spinous process, lamina, and articular process regions. Some of the lamina and root of the spinous process was removed with the osteotome, the contralateral lamina was undercut, and the medial part of the articular process was removed. Part of the intervertebral tissue was also removed along with the nucleus pulposus tissue. The cartilage endplate was scraped, both allogeneic and autologous bone particles were implanted in the vertebral space, then the fusion cage was implanted in the vertebral space (Weigao, Shandong, Co., China), and the size of the cage is 24–30 mm long, 12–14 mm wide and 10–12 mm high. The pedicle screw was inserted along the pedicle screw guide pin after exiting the endoscopic system. The C-arm was subjected to fluoroscopic examination again to confirm that the internal fixation position was good, the incision was cleaned and sutured, and a drainage tube was inserted.

### Clinical indicators

The Operation time, Blood loss, and Postop hospitalization were recorded. The Visual Analog Scale (VAS) score, as well as the Oswestry Disability Index (ODI) index, were used to assess the degree of low back pain and lumbar spine function of patients preop, 7 d postop, 3 mo postop, and at the last follow-up.

### Radiological indicators

**Fig. 2:**
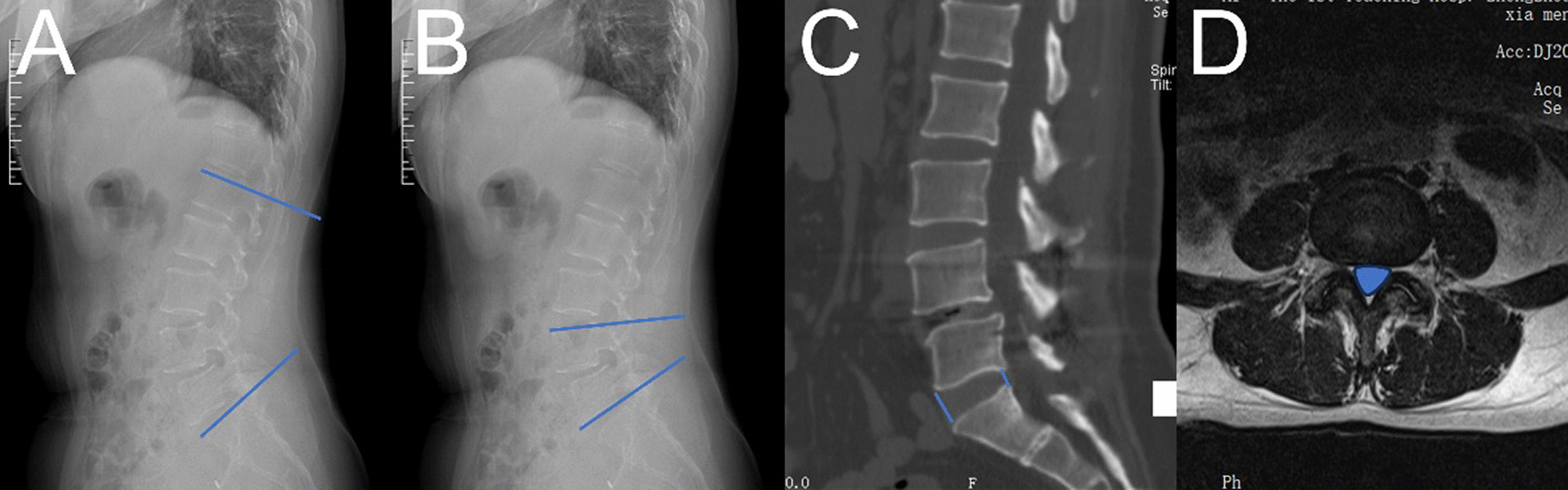
The measurement technique for each Radiological indicators. **A** Measurement of LL on lateral roentgenograph; **B** Measurement of Segmental angle on lateral roentgenograph; **C** Measurement of DH on sagittal CT; **D** Measurement of CSA of spinal canal on axial MRI

Preop DR, CT, and MRI of the lumbar spine were performed in all patients. The patients' Lumbar lordosis(LL), Segmental angle, the cross-sectional area(CSA) of the spinal canal, and Disk height (DH) were recorded at 7 d postop, 3 mo postop, and at the last follow-up (Fig. 2). The measurement method of DH adopts the average value of the sum of the heights of the anterior and posterior edges of intervertebral space. The fusion rate was determined by Bridwell's [9] fusion grading system at 3 mo postop, 6 mo postop and the last follow-up. All radiographic measurements were made by 2 independent observers, and the mean of the values was used for analysis. In the event of a discrepancy, a third senior reviewer was consulted.

### Statistical analysis

All statistical analyses were performed using the Statistical Package for the Social Sciences (SPSS) for Windows version 26.0 (IBM SPSS Statistics for Windows, Armonk, NY, USA). The continuous variables conforming to the normal distribution are measured as mean ± standard deviation. Independent *t*-tests were used for comparison with the same indicator group, and paired *t*-tests were used for comparison within the same indicator group. The count data are expressed in frequency, and the χ^2^-test was used to examine differences between the data of the two groups. *P* < 0.05 was considered statistically significant.

## Results

### General finding

A total of 107 patients were included in this study, 45 in the OLIF-AF group and 62 in the ULIF group. There was no statistical difference between the two groups in terms of gender, age, BMI, smoking, and follow-up time (*P* > 0.05) (Table 1).

**Table 1:** Basic information of patients in two groups

	OLIF-AF	ULIF	*P*
Sex			0.941
Male	20	28	
Female	25	34	
Average age (y)	59.43 ± 10.26	61.28 ± 9.41	0.452
BMI (kg/m^2^)	24.61 ± 3.14	25.46 ± 3.38	0.316
Smoke			0.959
Yes	22	30	
No	23	32	
Meyerding classification			0.640
I	26	33	
II	19	29	
Lesion level			0.663
L2/L3	9	8	
L3/L4	14	23	
L4/L5	22	31	
Follow-up (m)	26.62 ± 3.57	27.45 ± 4.96	0.341

Data presented as mean ± standard deviation

### Clinical efficacy evaluation

The operative time and Blood loss in OLIF-AF and ULIF groups were 108.23 ± 25.69 min versus 142.34 ± 35.81 min (*P* < 0.05); 63.49 ± 12.18 mL versus 91.23 ± 24.65 mL (*P* < 0.05); and there was no significant difference in Postop hospitalization between the two groups. (Table 2). The ODI at 7 d postop and 3 mo postop decreased from 56.91 ± 12.38 preoperatively to 28.23 ± 9.45 and 20.81 ± 8.32 (*P* < 0.05) in the OLIF-AF group and from 58.42 ± 13.25 to 36.51 ± 10.14 and 27.54 ± 9.13 (*P* < 0.05) in the ULIF group, but at the last follow-up (13.26 ± 6.72 vs. 17.41 ± 7.52, *P* = 0.121) the difference was not statistically significant. The VAS of low back pain was not statistically different between the two groups preop (*P* = 0.768), but at 7 d postop, 3 mo postop, and the last follow-up (3.51 ± 1.36 vs. 4.62 ± 1.51, 2.61 ± 1.24 vs. 3.41 ± 1.46, 1.34 ± 0.96 vs. 2.13 ± 1.17, *P* < 0.05), the results of OLIF-AF group were significantly superior to ULIF group. It is worth noting that there was no statistical difference between the two groups in terms of VAS scores for leg pain in preop, 7 d postop, 3 mo postop, and at the last follow-up (*P* > 0.05; Table 3).

**Table 2:** Comparison of perioperative condition between two groups

	OLIF-AF	ULIF	*P*
Operation time (min)	108.23 ± 25.69	142.34 ± 35.81	0.001
Blood loss (mL)	63.49 ± 12.18	91.23 ± 24.65	0.001
Postop hospitalization (d)	7.53 ± 2.18	7.82 ± 2.54	0.635

**Table 3:** Clinical efficacy evaluation of two groups

	OLIF-AF	ULIF	*P*
VAS of low back pain			
Preop	6.71 ± 1.89	6.58 ± 1.64	0.768
7 d postop	3.51 ± 1.36	4.62 ± 1.51	0.003
3 mo postop	2.61 ± 1.24	3.41 ± 1.46	0.027
Last follow-up	1.34 ± 0.96	2.13 ± 1.17	0.006
VAS of leg pain			
Preop	7.23 ± 2.14	6.92 ± 1.87	0.548
7 d postop	4.32 ± 1.51	4.48 ± 1.63	0.690
3 mo postop	2.94 ± 1.12	2.75 ± 1.36	0.558
Last follow-up	1.26 ± 0.83	1.43 ± 0.92	0.448
ODI (%)			
Preop	56.91 ± 12.38	58.42 ± 13.25	0.646
7 d postop	28.23 ± 9.45	36.51 ± 10.14	0.002
3 mo postop	20.81 ± 8.32	27.54 ± 9.13	0.004
Last follow-up	13.26 ± 6.72	17.41 ± 7.52	0.121

### Radiological result evaluation

In OLIF-AF group, LL increased from 36.29° ± 7.46° preop to 42.35° ± 8.21°, 43.61° ± 8.57°, and 41.29° ± 7.31° at 7 d postop, 3 mo postop, and at the last follow-up, with statistically significant differences (*P* < 0.05); However, ULIF group did not have this change, and at the same time OLIF-AF were significantly better than those of the ULIF group (*P* < 0.05; Table 4).

**Table 4:** Comparison of radiological parameters between the two groups

	OLIF-AF	ULIF	*P*
LL (°)			
Preop	36.29 ± 7.46	35.31 ± 6.72	0.581
7 d postop	42.35 ± 8.21^#^	37.25 ± 7.02	0.009*
3 mo postop	43.61 ± 8.57^#^	38.11 ± 7.36	0.007*
Last follow-up	41.29 ± 7.31^#^	36.54 ± 6.13	0.006*
Segmental angle (°)			
Preop	8.46 ± 2.31	8.19 ± 2.25	0.651
7 d postop	13.82 ± 2.65^#^	10.54 ± 2.41^#^	0.000*
3 mo postop	12.98 ± 2.04^#^	9.91 ± 1.57^#^	0.000*
Last follow-up	12.38 ± 1.71^#^	8.72 ± 1.38	0.000*
CSA of spinal canal (mm^2^)			
Preop	85.29 ± 16.15	89.91 ± 17.23	0.282
7 d postop	128.46 ± 23.54^#^	122.35 ± 21.42^#^	0.281
3 mo postop	131.91 ± 26.12^#^	121.85 ± 16.56^#^	0.058
Last follow-up	132.46 ± 24.81^#^	123.23 ± 17.24^#^	0.077
DH(mm)			
Preop	8.34 ± 2.05	8.27 ± 2.14	0.910
7 d postop	14.35 ± 3.69^#^	10.82 ± 3.23^#^	0.000*
3 mo postop	13.67 ± 3.21^#^	10.51 ± 2.98^#^	0.000*
Last follow-up	13.58 ± 2.83^#^	10.46 ± 2.31^#^	0.000*
Fusion (%, n)			
3 mo postop	55.56% (25)	30.64% (19)	0.010
6 mo postop	75.56% (34)	56.45% (35)	0.042
Last follow-up	93.33% (42)	91.94% (57)	0.917

^#^*P* < 0.05 means compared with Preop

*P < 0.05 means statistical significance between the two groups

Segmental angle in OLIF-AF group was 8.46° ± 2.31°, 13.82° ± 2.65°, 12.98° ± 2.04°, and 12.38° ± 1.71° preop, 7 d postop, 3 mo postop, and the last follow-up, with significant improvement postop compared to preop (*P* < 0.05); In ULIF group, there was a significant difference at 7 d postop and 3 mo postop compared to preop (*P* < 0.05). However, the results were not statistically different at the last follow-up (*P* = 0.086). The results of the OLIF-AF group were better than those of the ULIF group at the postop follow-up (*P* < 0.001; Table 4).

DH in OLIF-AF group increased from 8.34 ± 2.05 mm preop to 14.35 ± 3.69 mm at 7 d postop (*P* < 0.05), declined to 13.67 ± 3.21 mm at 3 mo postop (*P* = 0.484), and was 13.58 ± 2.83 mm at last follow-up (*P* = 0.303), representing an improvement rate of 62.83%; DH in ULIF group increased from 8.27 ± 2.14 preop to 10.82 ± 3.23 mm at 7 d postop (*P* < 0.05) and was maintained well at 3 mo postop (10.51 ± 2.98, *P* = 0.611) and last follow-up (10.46 ± 2.31, *P* = 0.481), with an improvement rate of 26.48%. The DH improvement rate was significantly higher in the OLIF-AF group than in the ULIF group throughout the follow-up period (*P* < 0.001) (Table 4).

The CSA of the spinal canal in the two groups improved from 85.29 ± 16.15 mm^2^ and 89.91 ± 17.23 mm^2^ preop to 128.46 ± 23.54 mm^2^ and 122.35 ± 21.42 mm^2^ at 7 d postop (*P* < 0.001) and stabilized, and at the last follow-up OLIF-AF group was 132.46 ± 24.81 mm^2^ with 55.31% expansion rate and 123.23 ± 17.24 mm^2^ with 37.10% expansion rate in ULIF group. There was no statistically significant difference between the two groups (*P* > 0.05) (Table 4).

The fusion rate at 3 mo postop was 55.56% (25/45) higher in the OLIF-AF group than in the ULIF group at 30.64% (19/62) (*P* = 0.010); the fusion rate at 6 mo postop was also significantly higher in OLIF-AF group than in ULIF group (*P* = 0.042). However, at Last follow-up, the fusion rate in the OLIF-AF group was slightly higher at 93.33% (42/45) than that in the ULIF group at 91.94% (57/62) (*P* = 0.917), the difference was not statistically significant (Table 4).

### Complications

The incidence of complications in the OLIF-AF group was 15.6% (7/45): 2 patient have endplate injury, but the patient did not show corresponding symptoms, and there was no fusion settling and good fusion in the subsequent follow-up; 2 patients had psoas muscle numbness combined with lateral thigh numbness, and 3 patient had left sympathetic trunk nerve injury. The symptoms completely disappeared 3 mo postop after routine guidance of functional exercise and nutrition nerve treatment.

The incidence rate of complications in the ULIF group was 16.1% (10/62): 3 cases had a dural laceration and 2 case had screw malposition, and no adverse sequelae were caused after immediate repair. Three patients had endplate injury and were instructed to delay postop ambulation; two patients developed radicular symptoms postop, and they recovered well after treatment such as improving circulation and nourishing nerves. There was no statistical difference in the incidence of complications between the two groups (*P* = 0.936).

### Discussion

With the concept of rapid rehabilitation gradually gaining popularity, various minimal LIF procedures have come into the view of spine surgeons, and currently, depending on the surgical access, the posterior approach ULIF as well as the lateral approach OLIF are more accepted and recognized. Previous posterior approach LIF has greatly affected the surgical outcome due to complications such as extensive paravertebral muscle injury, dural tears, and poor recovery of postoperative sagittal plane parameters [10]. While ULIF uses a dual-channel technique to greatly circumvent these complications, its recovery of the lumbar spine sequence is still poor. ALIF has the advantages of short operative time and minimal trauma; however, it has not been popularized due to its anatomical complexity and complications such as intraoperative injury to large abdominal vessels and abdominal organ damage ]11, 12]. Based on this, OLIF was developed. OLIF uses a retroperitoneal fat and lumbar major muscle gap approach, which perfectly avoids important neurovascular and is therefore gradually being promoted by spine surgeons.

Perioperative indicators are gradually coming into focus, and it has also been noted in the literature that prolonged operative time and increased blood loss have an impact on the increase in surgical complications. A recent review of 16 publications by Li et al. [13] reported a mean operative time of 95.2 min and blood loss of 109.9 ml for OLIF. Liu et al. [14] documented 60 patients and described a mean operative time of 5 h for ULIF, which was significantly longer than that of Song et al. [15] performed a retrospective study of 49 patients, which may be attributed to the operator's unskilled operation and steep initial learning curve. Chen et al. [16] have a retrospective analysis about 97 patients to assess the learning curve of the unilateral biportal endoscopic (UBE) technique for the treatment of single-level lumbar disk herniation by cumulative summation (CUSUM) method analysis. Making conclusions about 24 cases of single segmental UBE operation are needed to master the UBE technique. With the progress of the learning curve, the operative time decreased. However, the operative time was significantly longer than that of the OLIF group, which is also consistent with the results of the present study.

When the clinical outcomes of the two groups were analyzed, it was found that there was no difference between the two groups in terms of VAS of leg pain preop and after postop at each follow-up, but both groups showed significant improvement in postop compared to preop. However, for VAS of low back pain, OLIF-AF group was better than the ULIF group at 7 d postop, 3 mo postop, and at the last follow-up, probably because OLIF-AF did not require stripping of the posterior spinal muscles and did not require removal of bony structures such as the laminae, which resulted in the perfect preservation of the posterior spinal structures and reduced the incidence of peripheral tissue damage and medically induced neurological disorders [17]. This study is essentially a comparative analysis of the curative effects of direct decompression and indirect decompression. Hiyama et al. [18] analyzed the pain after direct decompression (minimally invasive—transforaminal interbody fusion, MIS-TLIF) and indirect decompression (extreme lateral interbody fusion, XLIF), the indirect decompression group had a more significant improvement in low back pain than the direct decompression group. Differences in the invasiveness of the procedures involving the posterior support elements may be responsible for the differences between groups, and this is consistent with the results in this study. When the ODI index was analyzed, it was found that both groups showed significant improvement at each postop follow-up compared to preop, with the OLIF-AF group showing better results than the ULIF group at 7 d and 3 mo postop; however, there was no statistical difference between the two groups at the final follow-up. This shows that the short-term efficacy of OLIF-AF is superior to ULIF. Zhao et al. [19] performed a retrospective analysis of 98 patients, of whom 46 were in the OLIF-AF group and 52 were in TLIF group obtained results consistent with our study. This may make OLIF-AF more consistent with the concept of accelerated recovery.

Additional findings confirm that the restoration of postoperative sagittal balance has an important impact on patient prognosis, particularly in terms of LL and DH. To better restore DH, the OLIF-AF group chose larger-size cage devices for reducing disk bulge as well as reducing hypertrophy of the ligamentum flavum and further release the Intervertebral foramen height [7, 20, 21]. However, the large size cage restores the DH with corresponding side effects, excessive stress may lead to endplate injury, especially in the upper endplate, as well as to persistent postoperative neurological symptoms due to excessive nerve root strain [22, 23]. In this study, the short-term radiological results of the two groups were analyzed, OLIF-AF group was superior to the ULIF group in terms of DH recovery, and the rate of improvement in the LL and Segmental angle was better in the OLIF-AF group compared to ULIF group, which may be related to the use of a larger size cage in OLIF-AF group. Eum et al. [24] conducted a retrospective study on 22 patients, all patients underwent extreme transforaminal lumbar interbody fusion (eXTLIF) in unilateral biportal endoscopy (UBE) and used a large profile fusion cage. The follow-up time was more than 6 months, and satisfactory results were obtained. You et al. [25] performed biportal endoscopic extraforaminal lumbar interbody fusion (BE-EFLIF) on 12 patients with lumbar degenerative diseases, and 3D-printed porous titanium cage with large footprints was used in the operation, DH, SL and LL recovered well in postop. Previous studies have confirmed that the improvement of sagittal plane parameters is closely related to the improvement of neurological symptoms in patients [26, 27]]. And our findings confirm the superiority of OLIF-AF in the recovery of sagittal plane parameters. The improvement of CSA of the spinal canal is increasingly coming to the attention of spine surgeons, and throughout the follow-up period in this study, OLIF-AF group showed a progressive increase in the canal area, while ULIF group reached a more stable level right in postop, and the same results have been reported in previous reports on other indirect decompression techniques, which may be caused by spontaneous retraction of the fibrous ring and ligamentum flavum and the placement of cages, etc. [28–30].

In our study, the fusion rate was significantly better in the OLIF-AF group than in the ULIF group at 3 mo postop and 6 mo postop, but the fusion rate was comparable between the two groups at Last follow-up, which suggests that postop intervertebral fusion was faster in OLIF-AF group. This may be due to the larger size cage in OLIF-AF group allowing for a larger contact area with the endplates, as well as the large space within the fusion device increasing the amount of intraoperative bone grafting [31]. In addition, some studies have suggested that pressure stimulation between the cage and the endplate contributes to gradual fusion, which may be another possible explanation [32]. When the incidence of complications was counted, it was found that there was no statistically significant difference between the two groups, and neither had serious consequences.

This study still has some limitations: (1) this is a retrospective study with a relatively limited cohort, and it is possible that some differences between the two groups may not be found; (2) the follow-up period is short, and a longer follow-up is needed later to validate our results; (3) the sample size is small and it is a single-center study, and a larger sample size and more institutions need to be involved to validate the efficacy of these two surgical modalities.

## Conclusions

Both OLIF-AF and ULIF achieved good short-term results in the treatment of DLS, and both surgical approaches are desirable. However, OLIF-AF has advantages over ULIF in terms of postoperative restoration of lumbar sagittal parameters and earlier intervertebral fusion. However, long-term follow-up and larger clinical studies are needed to confirm this result.

## Data Availability

The datasets used and/or analyzed during the current study are available from the corresponding author on reasonable request.

## Abbreviations

OLIF: Oblique lateral interbody fusion
ULIF: Unilateral biportal endoscopic lumbar interbody fusion
LIF: Lumbar interbody fusion
DLS: Degenerative lumbar spondylolisthesis
VAS: The Visual Analog Scale
ODI: The Oswestry Disability Index
CSA: The cross-sectional area
OLIF-AF: OLIF combined with anterolateral single screwrod fixation
LL: Lumbar lordosis
DH: Disk height
